# The *ITGAV *rs3738919-C allele is associated with rheumatoid arthritis in the European Caucasian population: a family-based study

**DOI:** 10.1186/ar2221

**Published:** 2007-07-03

**Authors:** Laurent Jacq, Sophie Garnier, Philippe Dieudé, Laëtitia Michou, Céline Pierlot, Paola Migliorini, Alejandro Balsa, René Westhovens, Pilar Barrera, Helena Alves, Carlos Vaz, Manuela Fernandes, Dora Pascual-Salcedo, Stefano Bombardieri, Jan Dequeker, Timothy R Radstake, Piet Van Riel, Leo van de Putte, Antonio Lopes-Vaz, Elodie Glikmans, Sandra Barbet, Sandra Lasbleiz, Isabelle Lemaire, Patrick Quillet, Pascal Hilliquin, Vitor Hugo Teixeira, Elisabeth Petit-Teixeira, Hamdi Mbarek, Bernard Prum, Thomas Bardin, François Cornélis, the European Consortium on Rheumatoid Arthritis Families

**Affiliations:** 1 GenHotel-EA3886, Evry-Paris VII Universities, Member of the AutoCure European Consortium, 2 rue Gaston Crémieux, 91057 Evry-Genopole cedex, France; 2 Centre Hospitalier Sud Francilien, 59 bd Henri Dunant, 91100 Corbeil-Essonnes, France; 3 Service de rhumatologie, Hôpital Bichat, AP-HP, 46 rue Henri Huchart, 75018 Paris, France; 4 Service de rhumatologie, Hôpital Lariboisière, AP-HP, 2 rue Ambroise Paré, 75010 Paris, France; 5 Pisa University, 56126 Pisa, Italy; 6 La Paz Hospital, 28046 Madrid, Spain; 7 Katholieke Universiteit Leuven, BE-3000 Leuven, Belgium; 8 Nijmegen University, 6500HB Nijmegen, The Netherlands; 9 Porto San Joao Hospital, 4200 Porto, Portugal; 10 Faculty of Medicine, University of Coimbra, Coimbra, Portugal; 11 Laboratoire Statistique et Génome, Genopole, Tour Evry 2, 91000 Evry, France; 12 Unité de Génétique Clinique, Hôpital Lariboisière, AP-HP, 2 rue Ambroise Paré, 75010 Paris, France

## Abstract

The integrin αvβ3, whose αv subunit is encoded by the *ITGAV *gene, plays a key role in angiogenesis. Hyperangiogenesis is involved in rheumatoid arthritis (RA) and the *ITGAV *gene is located in 2q31, one of the suggested RA susceptibility loci. Our aim was to test the *ITGAV *gene for association and linkage to RA in a family-based study from the European Caucasian population.

Two single nucleotide polymorphisms were genotyped by PCR-restriction fragment length polymorphism in 100 French Caucasian RA trio families (one RA patient and both parents), 100 other French families and 265 European families available for replication. The genetic analyses for association and linkage were performed using the comparison of allelic frequencies (affected family-based controls), the transmission disequilibrium test, and the genotype relative risk.

We observed a significant RA association for the C allele of rs3738919 in the first sample (affected family-based controls, RA index cases 66.5% versus controls 56.7%; *P = *0.04). The second sample showed the same trend, and the third sample again showed a significant RA association. When all sets were combined, the association was confirmed (affected family-based controls, RA index cases 64.6% versus controls 58.1%; *P = *0.005). The rs3738919-C allele was also linked to RA (transmission disequilibrium test, 56.5% versus50% of transmission; *P = *0.009) and the C-allele-containing genotype was more frequent in RA index cases than in controls (RA index cases 372 versus controls 339; *P = *0.002, odds ratio = 1.94, 95% confidence interval = 1.3–2.9).

The rs3738919-C allele of the *ITGAV *gene is associated with RA in the European Caucasian population, suggesting *ITGAV *as a new minor RA susceptibility gene.

## Introduction

Rheumatoid arthritis (RA) is the most common human systemic autoimmune disease (0.8% prevalence in the European Caucasian population), affecting women preferentially [1]. The disease is characterized by a chronic inflammation of the synovial tissues leading to the formation of the rheumatoid pannus, which erodes adjacent cartilage and bone, causing subsequent joint destruction. One hallmark of the pannus is hyperangiogenesis [2].

Previous studies have indicated that the risk of developing the disease in siblings of affected individuals is 2–17 times higher than in the general population, suggesting the importance of genetic factors [1]. Two RA genes have so far been established and confirmed using familial material, *HLA-DRB1 *and *PTPN22 *[3,4], but they account only for a part of the RA genetic component. The dense genome scan realized in our laboratory suggested 19 non-*HLA *regions in the French Caucasian population [5] and one of these, 2q31, contains the *ITGAV *gene (alias *CD51*, *αv*), which encodes the αv subunit of the integrin family. This family is composed of at least 24 heterodimeric combinations of 18 α subunits and nine β subunits. These transmembranous receptors are expressed at the surface of numerous cells (endothelial cells, macrophages, monocytes, osteoclasts, platelets) and recognize the RGD sequence (Arg–Gly–Asp) of many ligands (such as vitronectin, fibronectin, osteopontin, sialoprotein, thrombospondin, fibrinogen, von Willebrand factor, tenascin, agrin, matrix metalloproteinases, and prothrombin) [6]. The integrins are involved in several functions including adhesion of activated endothelial cells with the extracellular matrix, proliferation, migration, and differentiation signals of vascular cells [6].

The αvβ3 integrin is well documented to play a key role in angiogenesis, and the *ITGAV *knockout animal model is lethal *in utero *for 80% with a presence of large vascular anomalies [7,8].

Angiogenesis also plays a key role in RA when the synovial membrane becomes hyperplasic and destroys the cartilage. We can observe an excess of blood cells (macrophages, T lymphocytes) in the synovial membrane and fluid, and some αvβ3 ligands (that is, fibrinogen or osteopontin) are abundant in the RA synovial fluid [7]. Moreover, some proangiogenic mediators (that is, vascular endothelial growth factor) are overexpressed in RA synovial membrane and serum [9,10].

In addition, several αvβ3 antagonists and angiogenesis inhibitors have been successfully tested on RA animal models [11-14]. The αvβ3 integrin could therefore become a new therapeutic target in RA, and some clinical studies have already begun [15].

Our aim was to use RA familial material to test two intronic *ITGAV *single nucleotide polymorphisms (SNPs) for RA association and linkage in the European Caucasian population.

## Materials and methods

All subjects provided informed consent, and the ethics committee of Hôpital Bicêtre (Kremlin-Bicêtre, Assistance Publique-Hôpitaux de Paris, France) approved the study. RA families were recruited through a national media campaign followed by selection of individuals who fulfilled the 1987 American College of Rheumatology criteria for RA according to the physicians in charge of the patients [16]. A rheumatologist university fellow reviewed all clinical data.

### Sample 1

Sample 1 (Table 1) constituted the DNA from 100 French Caucasian unrelated trio families (one RA patient and both parents) with the four grandparents of French Caucasian origin. Among these 100 RA patients, 87 were women; their mean age at disease onset was 32 years. In total, 81 patients were rheumatoid factor positive, 78 patients carried at least one *HLA-DRB1 *'shared epitope' susceptibility allele (DRB1*0101, DRB1*0102, DRB1*0401, DRB1*0404, DRB1*0405, DRB1*0408, DRB1*1001) [17] and 90 patients presented erosion.

**Table 1:** Characteristics of rheumatoid arthritis (RA) index cases from the investigated samples

	Sample 1 (*n *= 100)	Sample 2 (*n *= 100)	Sample 3 (*n *= 265)
Females (%)	87	90	86
Mean age of disease onset (years) (±standard deviation)	32 (±10)	31 (±6)	30 (±9)
Mean disease duration (years) (±standard deviation)	18 (±7)	16 (±8)	8 (±7)
RA patients with bone erosions (%)	90	79	72
RA patients seropositive for rheumatoid factor (%)	81	76	73
RA patients carrying at least one *HLA-DRB1 *shared epitope allele (%)	78	80	Not available

*n*, number of cases.

### Sample 2

Sample 2 (Table 1) was made up of the DNA from another 100 French Caucasian unrelated trio families with the same characteristics as sample 1. Among these 100 RA patients, 90 patients were women; their mean age at disease onset was 31 years. In all, 76 patients were rheumatoid factor positive, 80 patients carried at least one *HLA-DRB1 *shared epitope and 79 patients had an erosive disease.

### Sample 3

Sample 3 (Table 1) contained the DNA from 265 European Caucasian unrelated trio families with the same characteristics as sample 1, except for a shorter mean disease duration and a different ethnic origin (Caucasian families from France, Italy, Portugal, Spain, Belgium, and The Netherlands).

### Genotyping

DNA was isolated and purified from whole blood according to standard protocols [18]. Two intronic SNPs were selected at the 5' and 3' ends of the gene with a minor allele frequency >25% for European population databases. Moreover the presence of a restriction site for one of the alleles was required (SNP1, rs3768777; SNP2, rs3738919 [19,20]). Genotyping was performed by the PCR followed by restriction fragment length polymorphism method [21].

The designed primers were: sense, 5'-AAGTTGCCAACGTTCCGCGTTGCA-3' and antisense, 5'-GTAGTAGAAGATGGTCCTATCCACG-3' for SNP1; and sense, 5'-ATTTCCAGGTGGAACTTCTTTTGGA-3' and antisense, 5'-TCACAATTCAGATTTTTGCCACTGG-3' for SNP2.

PCR amplification of SNP1 and SNP2 was performed on each sample in a 25 μl reaction volume consisting of 10 U PCR buffer (Perkin Elmer, Boston, MA, USA), 1.25 mM each dNTP, 1.25 U AmpliTaq Gold DNA polymerase (Applied Biosystems, Foster City, CA, USA), 3 mM MgCl_2_, 0.0125 nM of the two primers and 50 ng genomic DNA, diluted to the final volume with H_2_O on an Eppendorf thermocycler using a hot start procedure. The PCR program was carried out using a first denaturation cycle of 94°C for 10 minutes followed by 37 cycles of denaturation at 94°C for 40 seconds, with an annealing temperature at 67°C for 30 seconds followed by an elongation step at 72°C for 1 minute. One final cycle of the extension was performed at 72°C for 2 minutes.

For SNP1, a 341-bp amplified fragment was digested with *Nla*III, generating two fragments when the restriction site was present (A allele). For SNP2, the resulting 501-bp fragment was digested with *Alu*I, generating three fragments for the C allele (126 bp, 161 bp and 214 bp), and two fragments for the A allele (permanent restriction site allowing one to validate the restriction protocol; 161 bp and 340 bp). Genotypes were assessed blindly by two independent investigators (LJ and CP). CEPH controls (1347-02 and 884-15) and 40 patients chosen at random were genotyped for quality control. All genotype data will be available online [22].

### Power calculation

Using the European population minor allele frequency of 29% and 35% for SNP1 and SNP2, respectively, a sample size of 100 patients and 100 controls, and the arc sinus transformation method described by Garnier and colleagues [23], we had 80% power to detect an association (*P *< 0.05) if the difference in allelic frequencies between patients and controls was at least 11% for SNP1 and 12.2% for SNP2.

### Statistical analysis

Prior to association tests, we checked the Hardy–Weinberg equilibrium in 'virtual controls' (constituted by parental untransmitted alleles to RA index cases).

The association and linkage between each polymorphism and RA was examined by three different methods: the affected family-based controls (AFBAC) method was used to compare transmitted and untransmitted allelic frequencies across all families, the transmission disequilibrium test (TDT) was used to detect linkage through preferential transmission of one allele to the affected subjects, and the genotype relative risk (GRR) test was used to compare the genotypic distribution in patients and controls [24-26]. The significance of the *P *value was assessed at 5%, leading to replication tests in sample 2 and, in the case of relevant results, in the larger sample 3.

## Results

### Hardy–Weinberg equilibrium

Hardy–Weinberg equilibrium in the virtual controls was respected for SNP1 and SNP2 in sample 1 and in the replication samples (data not shown).

### Test for association and linkage in sample 1

We observed neither significant association nor linkage between SNP1 and RA in sample 1. For SNP2, we observed a significant association for the C allele and a strong trend for a RA linkage (AFBAC, RA index cases 66.5% versus controls 56.7%, *P = *0.04; TDT, 59.7% of transmission versus 50%, *P *= 0.06) (Table 2). The GRR test showed a significant increase of the C/C genotype and an excess of C-allele-containing genotypes in patients (Table 3).

**Table 2:** Affected family-based control and transmission disequilibrium test analyses for single nucleotide polymorphism (SNP)1 and SNP2 in sample 1 of rheumatoid arthritis trio families

Allele	Affected family-based controls			Transmission disequilibrium test		
	
	Rheumatoid arthritis cases	Controls	*P*	Transmission (%)	*n*	*P*
SNP1						
A	0.360	0.320	0.39	54.4	90	0.39
G	0.640	0.680				
SNP2						
C	0.665	0.567	0.04	59.7	92	0.06
A	0.335	0.433				

*n*, number of heterozygote parents.

**Table 3:** Genotype relative risk analysis for single nucleotide polymorphism (SNP)1 and SNP2 in sample 1 of rheumatoid arthritis trio families

Genotype	Rheumatoid arthritis cases	Controls	*P*
SNP1			
A/A	16	7	0.1 (global)
A/G	40	50	
G/G	44	43	
SNP2			
C/C	45	32	0.03 (C/C versus C/A + A/A)
C/A	38	46	0.36 (C/C + C/A versus A/A)
A/A	13	18	

The linkage disequilibrium test showed a weak linkage disequilibrium between SNP1 and SNP2 (*D' *= 0.33), and were thus considered independent. The results of the haplotypic TDT analysis showed a significant undertransmission of the SNP1/SNP2 *GA *haplotype (21 versus 37, *P = *0.03), and a trend for an overtransmission of the two haplotypes containing the C allele of SNP2 (data not shown).

When stratifying the sample for the families with the index presenting at least one *PTPN22-620W *allele or the *HLA-DRB1 *allele shared epitope status, no correlation with the *ITGAV *genotypes could be observed (data not shown).

### Test for association and linkage in sample 2

The significant association observed for SNP2 in sample 1 led to a replication test in a second set of 100 French Caucasian Trio families (sample 2) on the hypothesis of an association of the C allele.

In this sample, we observed a trend for association and linkage of the C allele with RA (AFBAC, RA index cases 63.1% versus controls 59.6%, *P = *0.4; TDT, 52.6% of transmission, *P = *0.6) (Table 4). The GRR test showed a trend for an excess of the C-allele-containing genotype in RA index cases compared with controls (90 RA index cases versus 79 controls, *P = *0.09) but not for the C/C genotype (Table 5).

**Table 4:** Affected family-based control and transmission disequilibrium test analyses for single nucleotide polymorphism 2 in sample 2 of rheumatoid arthritis trio families

Allele	Affected family-based controls			Transmission disequilibrium test		
	
	Rheumatoid arthritis cases	Controls	*P*	Transmission (%)	*n*	*P*
C	0.631	0.596	0.4	52.6	95	0.6

*n*, number of heterozygote parents.

**Table 5:** Genotype relative risk analysis for single nucleotide polymorphism 2 in sample 2 of rheumatoid arthritis trio families

Genotype	Rheumatoid arthritis cases	Controls	*P*
C/C	33	39	0.4 (C/C versus C/A + A/A)
C/A	57	40	0.09 (C/C + C/A versus A/A)
A/A	8	19	

The combination of the two samples, authorized by the absence of any significant clinical difference between them, showed a marginally significant association of the C allele (AFBAC, RA index cases 64.8% versus controls 58.2%, *P = *0.05; TDT, 56.1% of transmission, *P = *0.09) and a significant excess of the C-allele-containing genotype in RA index cases compared with controls (173 RA index cases versus 157 controls, *P = *0.02).

### Test for association and linkage in sample 3

The trend for association of the C allele observed in sample 2 was in the same direction as the significant association observed in sample 1, without reaching statistical significance – notably due to a lack of power (the power to detect a significant association in sample 2, based on the allelic frequencies in sample 1, with *P *< 0.05, was only 51%). A larger replication test (265 families, sample 3) was therefore conducted on the hypothesis of an association of the C allele and of the C-allele-containing genotype.

We observed a significant RA association and linkage for the C allele (AFBAC, RA index cases 64.4% versus controls 57.8%, *P = *0.03; TDT, 57% of transmission versus 50%, *P *= 0.04) (Table 6). This increase was supported by a significant increase of the C-allele-containing genotype in patients (199 RA index cases versus 182 controls, *P = *0.02) (Table 7).

**Table 6:** Affected family-based control and transmission disequilibrium test analyses for single nucleotide polymorphism 2 in sample 3 of rheumatoid arthritis trio families

Allele	Affected family-based controls			Transmission disequilibrium test		
	
	Rheumatoid arthritis cases	Controls	*P*	Transmission (%)	*n*	*P*
C	0.644	0.578	0.03	57	200	0.04

*n*, number of heterozygote parents.

**Table 7:** Genotype relative risk analysis for single nucleotide polymorphism 2 in sample 3 of rheumatoid arthritis trio families

Genotype	Rheumatoid arthritis cases	Controls	*P*
C/C	88	76	0.2 (C/C versus C/A + A/A)
C/A	111	106	0.02 (C/C + C/A versus A/A)
A/A	22	39	

### Test for association and linkage in the combined samples 1 + 2 + 3

The combination of the three samples, authorized by the absence of a significant clinical difference between them, confirmed association and linkage for the C allele (AFBAC, 64.6% versus 58.1%, *P = *0.005; TDT, 56.5% of transmission, *P = *0.009) (Table 8). The GRR test showed an excess of the C-allele-containing genotype in patients (372 RA index cases versus 339 controls, *P = *0.002, odds ratio = 1.94, 95% confidence interval = 1.3–2.9) (Table 9).

**Table 8:** Affected family-based control and transmission disequilibrium test analyses for single nucleotide polymorphism 2 in the combined samples 1 + 2 + 3

Allele	Affected family-based controls			Transmission disequilibrium test		
	
	Rheumatoid arthritis cases	Controls	*P*	Transmission (%)	*n*	*P*
C	0.646	0.581	0.005	56.5	387	0.009

*n*, number of heterozygote parents.

**Table 9:** Genotype relative risk analysis for single nucleotide polymorphism 2 in the combined samples 1 + 2 + 3

Genotype	Rheumatoid arthritis cases	Controls	*P*
C/C	166	148	0.1 (C/C versus C/A + A/A)
C/A	206	191	0.002 (C/C + C/A versus A/A)
A/A	43	76	

## Discussion

We studied the *ITGAV *gene, a good RA candidate gene for its function implicated in angiogenesis, and its chromosomal location (in one of the 19 suggested non-*HLA *loci of our dense genome scan) [5]. We observed a significant RA association for the C allele of rs3738919 in a first sample of French Caucasian families, the same trend in replication sample 2, and again a significant association in replication sample 3 (European Caucasian families). Finally, significant RA association and linkage were observed when all sets were combined.

The association and linkage evidences provided by the present study remain nevertheless statistically modest, suggesting at most a minor RA susceptibility marker. Further studies in independent samples will be needed to definitively establish association and linkage of the *ITGAV rs3738919-C *allele to RA. For the observed allelic frequencies of 64.6% in patients versus 58.1% in controls, a sample size of 350 families would be required to obtain, with 80% power (*P *< 0.05), an independent replication of the association evidence reported here.

Once this association had been replicated, resequencing would be necessary to identify exonic and promoter SNPs to refine the associated haplotype.

In the same way, the chromosome 2 linkage suggestion observed in the genome scan of our laboratory could not be totally explained by the findings of the *ITGAV *linkage; hence, with the overtransmission observed in the TDT, the allele sharing expected for the affected sib-pair siblings would be about 53% and would necessitate thousands of sibling pairs to be revealed. Other RA genes in this chromosomal location and/or epistatic effects could be expected to be stronger RA factors that remain to be discovered.

Since the association evidence is modest, no genetic testing would be clinically indicated. Instead, the clinical relevance of the finding is likely to come through better understanding of the RA pathophysiology and may lead to new therapeutic targets.

Contrary to the result of the GRR test in sample 1, which suggested a recessive effect of the *ITGAV rs3738919-C *allele, the result of the larger combined sample is more in favour of a dominant effect of this marker. This difference could be explained by the relatively small size of the first sample.

Finally, regarding the key function of angiogenesis in others diseases, and in particular in cancers, it would be interesting to test the *ITGAV rs3738919-C *allele in these phenotypes.

## Conclusion

The present study showed a significant association and linkage for the *rs3738919-C *allele of the *ITGAV *gene with RA in the European Caucasian population, suggesting *ITGAV *as a new minor RA susceptibility gene in this population.

## Abbreviations

AFBAC = affected family-based controls; bp = base pair; GRR = genotype relative risk; PCR = polymerase chain reaction; RA = rheumatoid arthritis; SNP = single nucleotide polymorphism; TDT = transmission disequilibrium test.

## Competing interests

The authors declare that they have no competing interests.

## Authors' contributions

LJ, CP, EG and SG carried out the molecular genetic studies. LJ, CP, SBa, SG, PD, LM, HM, VHT, BP, EP-T and FC performed acquisition and analysis of the data. LM, SL, IL, PQ, PH, PM, AB, RW, PB, HA, CV, MF, DP-S, SBo, JD, TRR, PVR, LvdP, AL-V, TB, and the European Consortium on Rheumatoid Arthritis Families contributed to the recruitment of families and to the acquisition of clinical data. All authors read and approved the final manuscript.
